# Association of Child Growth Failure Indicators With Household Sanitation Practices in India (1998-2021): Spatiotemporal Observational Study

**DOI:** 10.2196/41567

**Published:** 2024-05-24

**Authors:** Lovely Jain, Sreya Pradhan, Arun Aggarwal, Bijaya Kumar Padhi, Ramaiah Itumalla, Mahalaqua Nazli Khatib, Shilpa Gaidhane, Quazi Syed Zahiruddin, Celso Augusto Guimarães Santos, Khalid AL-Mugheed, Tahani Alrahbeni, Neelima Kukreti, Prakasini Satapathy, Sarvesh Rustagi, Petra Heidler, Roy Rillera Marzo

**Affiliations:** 1 Department of Community Medicine and School of Public Health Postgraduate Institute of Medical Education and Research Chandigarh India; 2 IPE Global House Delhi India; 3 School of Management The Apollo University Chittoor India; 4 Division of Evidence Synthesis, Global Consortium of Public Health and Research Datta Meghe Institute of Higher Education Wardha India; 5 One Health Centre, Jawaharlal Nehru Medical College Datta Meghe Institute of Higher Education Wardha India; 6 Global Health Academy, Division of Evidence Synthesis, School of Epidemiology and Public Health and Research, Jawaharlal Nehru Medical College Datta Meghe Institute of Higher Education and Research Wardha India; 7 Department of Civil and Environmental Engineering, Federal University of Paraíba João Pessoa Brazil; 8 Adult Health Nursing and Critical Care Riyadh Elm University Riyadh Saudi Arabia; 9 Molecular Toxicology and Genetics Riyadh Elm University Riyadh Saudi Arabia; 10 School of Pharmacy Graphic Era Hill University Dehradun India; 11 Center for Global Health Research, Saveetha Medical College and Hospital, Saveetha Institute of Medical and Technical Sciences Saveetha University Chennai India; 12 Medical Laboratories Techniques Department AL-Mustaqbal University Hillah, Babil Iraq; 13 School of Applied and Life Sciences, Uttaranchal University Dehradun India; 14 Institute International Trade and Sustainable Economy IMC Krems University of Applied Sciences Krems Austria; 15 Faculty of Humanities and Health Sciences Curtin University Miri Sarawak Malaysia

**Keywords:** undernutrition, malnutrition, stunting, wasting, underweight, sanitation, WaSH, LISA, NFHS, DHS, spatial epidemiology, epidemiology, children, child, India, intervention

## Abstract

**Background:**

Undernutrition among children younger than 5 years is a subtle indicator of a country’s health and economic status. Despite substantial macroeconomic progress in India, undernutrition remains a significant burden with geographical variations, compounded by poor access to water, sanitation, and hygiene services.

**Objective:**

This study aimed to explore the spatial trends of child growth failure (CGF) indicators and their association with household sanitation practices in India.

**Methods:**

We used data from the Indian Demographic and Health Surveys spanning 1998-2021. District-level CGF indicators (stunting, wasting, and underweight) were cross-referenced with sanitation and sociodemographic characteristics. Global Moran I and Local Indicator of Spatial Association were used to detect spatial clustering of the indicators. Spatial regression models were used to evaluate the significant determinants of CGF indicators.

**Results:**

Our study showed a decreasing trend in stunting (44.9%-38.4%) and underweight (46.7%-35.7%) but an increasing prevalence of wasting (15.7%-21.0%) over 15 years. The positive values of Moran I between 1998 and 2021 indicate the presence of spatial autocorrelation. Geographic clustering was consistently observed in the states of Madhya Pradesh, Jharkhand, Odisha, Uttar Pradesh, Chhattisgarh, West Bengal, Rajasthan, Bihar, and Gujarat. Improved sanitation facilities, a higher wealth index, and advanced maternal education status showed a significant association in reducing stunting. Relative risk maps identified hotspots of CGF health outcomes, which could be targeted for future interventions.

**Conclusions:**

Despite numerous policies and programs, malnutrition remains a concern. Its multifaceted causes demand coordinated and sustained interventions that go above and beyond the usual. Identifying hotspot locations will aid in developing control methods for achieving objectives in target areas.

## Introduction

Child malnutrition continues to pose a significant challenge in low- and middle-income countries. Around 60% of deaths among children younger than 5 years in these countries are attributable to malnutrition [1]. Much of this burden is seen in regions of South Asia, where 2 of every 5 stunted children globally are found [2]. An alarming irony lies in the fact that regions in South Asia such as India, Pakistan, and Bangladesh report child malnutrition rates that are higher than those in sub-Saharan Africa [3]. From the earliest Demographic and Health Surveys (DHS) in the 1990s to the most recent surveys in 2021, all countries have experienced a reduction in the prevalence of stunting. However, India and Pakistan recorded the lowest reductions—2.3 and 0.6 percentage points per year, respectively—compared with Bangladesh and Nepal, which recorded the largest reductions—2.9 and 4.1 percentage points per year, respectively. The Global Hunger Index, a comprehensive instrument used for measuring and monitoring hunger on a global, regional, and national scale, in 2022 ranked India 107th out of the 121 countries; additionally, India has recorded the highest rate of child wasting and has performed comparatively worse than its neighboring countries, with Pakistan scoring 99, Bangladesh scoring 84, Nepal scoring 81, and Sri Lanka scoring 64 [4]. Malnutrition is the leading cause of significant health and developmental issues among children globally, accounting for 3.5 million deaths and 35% of morbidities among children younger than 5 years [5]. For those who survive, malnutrition can cause lasting damage, increase the likelihood of child morbidity, contribute to poor cognitive development in childhood, result in short stature in adulthood, decrease adult productivity, increase the risk of perinatal and neonatal death for women, and heighten the risk for chronic disease if accompanied by overweight in later childhood [6]. Malnutrition bears noteworthy economic ramifications, as India experiences an annual loss of 4% of its gross domestic product due to malnutrition [7].

Childhood malnutrition is a complex issue. Merely increasing household income will not suffice to alleviate the childhood malnutrition if children lack food security, health care, education, water, and sanitation access [8]. According to a recent study, 3 key drivers of malnutrition include poor diet among children in their first years of life, dietary inadequacy among women before and during the pregnancy, and poor sanitation practices at both household and community levels [6,9]. A recent study based on large-scale national representative survey data of India found that the likelihood of stunting increased with decreased regional water availability, unimproved sanitation, and unsanitary stool disposal methods. Wasting was also exacerbated by a decline in regional water supplies and inadequate sanitation [7]. Household water, sanitation, and hygiene facilities and practices were reported as key mediating factors in the reduction of stunting in the Democratic Republic of Congo [9]. Children with better toilet facilities had a 37% less chance of being wasted [10]. Conversely, poor sanitation facilities could put the child at risk for a variety of infections and health problems [11]. Despite these issues being primarily associated with inadequate feeding patterns [12], addressing them remains challenging, particularly in India, where malnutrition affects both well-nourished and malnourished individuals [13]. According to the India State-Level Disease Burden Initiative, child growth failure (CGF) is responsible for almost one-fifth of all under-5 mortality in India, with a prevalence rate for stunting ranging from 21.3% to 49%, for wasting from 6.3% to 19.3%, and for underweight from 16.5% to 42.2% in 2017, showing a widespread geographical variance [14]. This disparity among Indian states makes achieving the Sustainable Development Goal 2 more challenging [15].

India has several nutrition-specific (food fortification and deworming) and nutrition-sensitive (Public Distribution System, Midday Meal Scheme, and POSHAN Abhiyaan) policies and programs in place to address recognized causes of malnutrition and child development. However, these programs encounter a variety of implementation challenges [16] across states, including poor targeting, leakage [17], inadequate infrastructure [18], corruption, and delayed payments [19]. If these large-scale social safety programs aim to address the fundamental drivers of hunger, it is necessary to bridge these implementation gaps. The provision of safe water and basic sanitation facilities in India has increased, although there remain disparities as indicated by the data reported by Water.org. According to recent statistics, a notable proportion of the Indian populace, specifically 6%, are deprived of access to potable water, whereas a significant majority (54%) lack access to properly managed household sanitation amenities. The score of India in terms of the availability of clean water and sanitation also reduced from 88 (2019) to 83 (2020) [20,21]. To gain a more nuanced understanding of the regional distribution of these indicators in India, a spatial-regional approach is needed. Additionally, to identify the determining factors holistically and particularly across spatial-regional contexts, this study used a spatial regression model to explain the determinants of the unequal distribution of stunting, wasting, and underweight. Pursuant to this recommendation, our research aims to present the spatiotemporal trends of CGF indicators (wasting, stunting, and underweight) among Indian children younger than 5 years from 1992 to 2020, as well as their correlation with household sanitation practices.

## Methods

### Overview

This study used data from Indian Demographic and Health Surveys, also known as the National Family Health Surveys (NFHS). The NFHS is a large-scale, multiround survey conducted in a representative sample of households across India. It used a stratified random sampling method at multiple stages, distinguishing between urban and rural areas as natural strata. The survey captures national and state-level data on fertility, infant and child mortality, the practice of family planning, maternal and child health, reproductive health, nutritional status, and use and quality of health and family planning services [22].

A total of 33,026, 51,555, 259,627, and 232,920 children younger than 5 years who participated in NFHS-2 (1998), NFHS-3 (2005), NFHS-4 (2015), and NFHS-5 (2021), respectively, were included in the analysis.

### Ethical Considerations

The study is based on a secondary data set from an NFHS survey that contains no personally identifying information about the survey participants. These NFHS data sets can be downloaded from the DHS program website after registration [23]. The NFHS survey had received ethical clearance from the International Institute for Population Science’s ethical review board before the survey. Additionally, the study protocol was reviewed and approved by the institutional research ethics committee of the Post Graduate Institute of Medical Education and Research, Chandigarh, India (vide letter number: NK/7176/MPH/188, July 27, 2021).

### Outcome Variables

The nutritional status of children served as the outcome variable in this study. Children with a height-for-age *z* score of less than −2 SD from the reference population median are considered stunted. Those with a height-for-age *z* score of less than −3 SD from the reference population median are classified as severely stunted [24]. Children with a weight-for-height *z* score of less than −2 SD from the reference population median are considered thin (wasted) or acutely undernourished. Those with a weight-for-height *z* score of less than −3 SD from the reference population median are classified as severely wasted [24]. Underweight children have a weight-for-age *z* score of less than −2 SD from the reference population median. Children are considered severely underweight if their weight-for-age *z* score is less than −3 SD from the median [24]. Height and age SD, weight and height SD, and weight and age SD data were provided for these indicators. Anthropometric *z* scores were calculated based on the World Health Organization (WHO) growth standards of children, as guided by the *DHS-7 Guide to DHS Statistics*. Children with height-for-age, weight-for-age, or weight-for-height *z* scores falling outside of the acceptable range were identified as having invalid data.

### Exposure Variable

We explored various characteristics that influence the outcome variables, as reported by previous studies [25,26]. The independent variables, along with potential confounding factors, included household sanitation facilities and sources of drinking water. Personal characteristics of children (eg, age and gender), maternal-related factors (eg, mother's education), and household-related factors (eg, wealth index and ethnicity [caste]) were among these influences. Water supply and sanitation facilities, as defined by the DHS, were divided into 2 categories: improved and unimproved. Households with improved drinking water sources included those with piped water to yard, plot, or dwelling; tube well or borehole; public tap or standpipe; rainwater; protected well; protected spring; boiled water; and reverse osmosis plant. Households with unimproved water sources included those using surface water (dam, river, lake, canal, stream, or pond and irrigation), unprotected springs, unprotected wells, tanker trucks, or carts with a small tank.

Improved sanitation facilities comprised flush or pour-flush to piped sewer systems, septic tanks, pit latrines, twin pits, pit latrines with slabs, ventilated improved pit or biogas latrines, and composting toilets. Unimproved sanitation facilities included any type of unimproved toilet facility, shared facilities, and open defecation (no facility, bush, or field).

### Data Extraction

**Figure 1:**
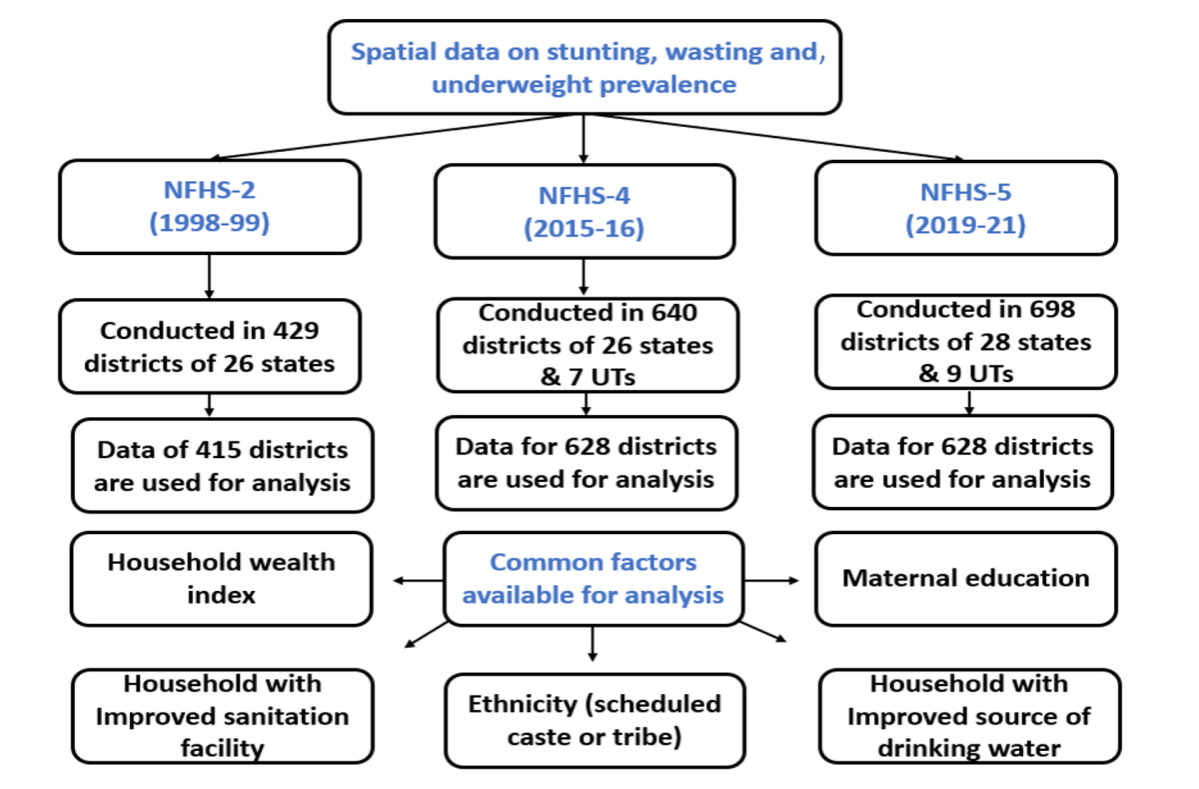
Framework deployed in data extraction and analysis in this study. NFHS, National Family Health Surveys; UT, union territory.

Figure 1 illustrates the framework used in the data extraction and analysis for this study. The data set, presented in the Stata(.dta) file format, was extracted from the DHS survey data set files. The extracted data were cleaned for the variables of interest using Stata (StataCorp). Data from NFHS-2, NFHS-3, NFHS-4, and NFHS-5 were used for the trend analysis.

### Data Analysis

#### Spatial Analysis

**Figure 2:**
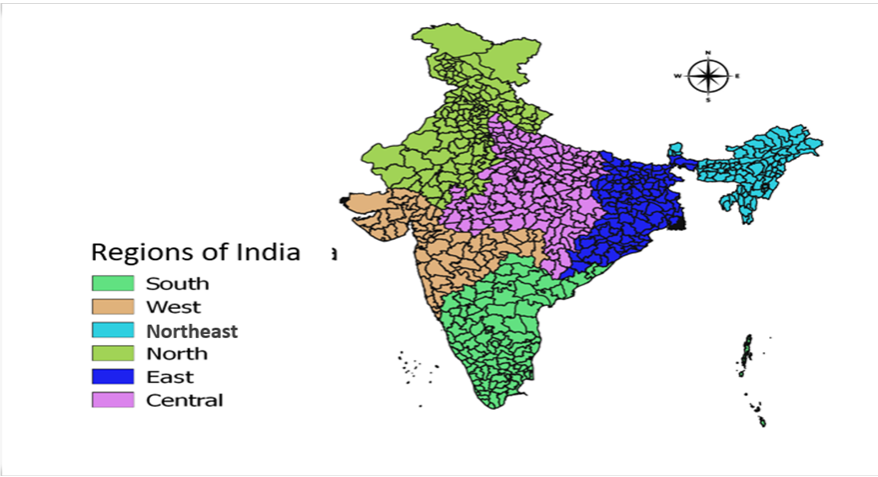
Regions of India.

We used India’s 2011 Census shapefile, which includes 640 districts for spatial analysis using the district-level data from NFHS-2, NFHS-4, and NFHS-5 (Figure 2).

#### Exploratory Spatial Analysis

The spatial autocorrelation statistic (Global Moran I) was used to assess the clustering or randomness of data, that is, whether the CGF patterns are randomly distributed, scattered, or clustered. If the Moran I value is closest to –1, the prevalence of wasting, stunting, and underweight is distributed; if the Moran I value is closest to +1, these variables are clustered in the study region. A Moran I value of zero, however, indicates a random distribution of CGF indications. Once it was confirmed that the distribution of stunting, wasting, and underweight is nonrandom, local Moran I was used to study the local-level cluster locations of CGF indication in India using the LISA (Local Indicator of Spatial Association) cluster map [27].

Hotspot clusters and cold spot clusters were identified by the local Moran I. Hotspots (High-High) and cold spots (Low-Low) were identified. It also accounted for outliers where high values were mostly surrounded by low values (High-Low) and outliers where the low values were primarily surrounded by high values (Low-High) [28]. The bivariate LISA was used to calculate the relationship between independent variables and CGF indicators.

The LISA significance map was used for hotspot analysis, computing the *P* value for the estimation of the statistical significance of the clustering area. The *P* value associated with 99%, 95%, and 90% confidence levels was used in this study to determine the presence of substantial clustering. The software used in this study included GeoDa 1.18 (Spatial Analysis Laboratory of the University of Illinois at Urbana-Champaign) [29] and QGIS 2.18.16 (QGIS Development Team) [30].

#### Confirmatory Spatial Analysis

The regression technique was used to investigate the relationship between the percentage of stunting, wasting, and underweight and background characteristics (improved and unimproved drinking water supply and toilet facilities, maternal education, ethnicity [caste], and wealth index). The choice of the regression technique was determined by the presence or absence of multicollinearity, homoscedasticity (constant variance), and normal distribution in the data. To check if the explanatory variables correspond, the multicollinearity condition number was used. Homoscedasticity was determined using the Breusch-Pagan test. The Jarque-Bera test was used to assess the normality of the error distribution. If the independent variables were found to be uncorrelated, followed a normal distribution, and had a constant variance (homoscedasticity), the ordinary least-squares regression, a nonspatial regression technique, would be used. If this assumption was broken, the use of spatial regression would be justified. Spatial regression is used for predicting the value of the dependent variable based on the collection of values from independent factors while considering spatial dependence. Spatial lag and spatial error are 2 types of spatial dependence. The spatial lag model assumes that the result of the outcome variable is affected by neighboring areas, whereas the spatial error model evaluates the impact of variables that were not included in the regression model but have an impact on the dependent variable [31]. The key difference between the 2 models is the inclusion of error terms of spatial dependence in the spatial error model. The analysis was conducted with the assistance of the RStudio [32] program.

## Results

### Characteristics

**Figure 3:**
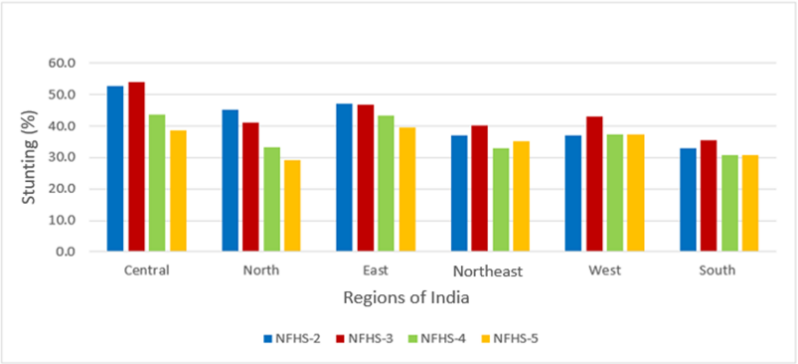
Trends of stunting by region; data from Indian Demographic and Health Surveys 1998, 2005, 2015, and 2021. NFHS: National Family Health Survey.

**Figure 4:**
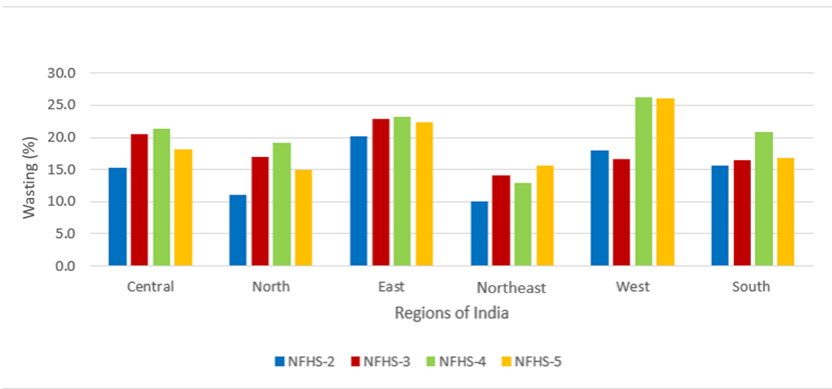
Trends of wasting by region; data from Indian Demographic and Health Surveys 1998, 2005, 2015, and 2021. NFHS: National Family Health Survey.

**Figure 5:**
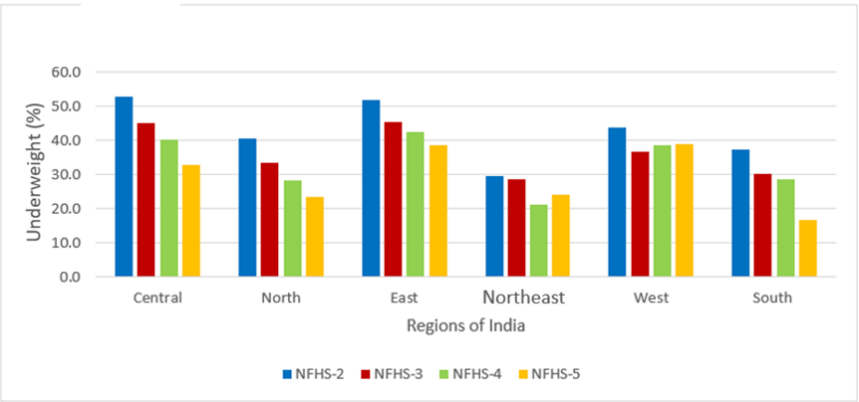
Trends of underweight by region; data from Indian Demographic and Health Surveys 1998, 2005, 2015, and 2021. NFHS: National Family Health Survey.

A total of 24,395, 45,377, 215,511, and 206,112 observations were available from the survey rounds 1998-1999, 2005-2006, 2015-2016, and 2019-2021, respectively. Table 1 describes the sociodemographic characteristics of the samples from NFHS-2, NFHS-3, NFHS-4, and NFHS-5 that were analyzed. Most of the sociodemographic distribution in the sample remained almost the same across NFHS-3, NFHS-4, and NFHS-5. However, in NFHS-2, there was higher representativeness of children from 0 to 24 months, particularly in middle wealth quintile; around 50% of mothers of children were uneducated in both NFHS-2 and NFHS-3, 30% in NFHS-4, and 21% in NFHS-5. There were 22%, 13%, 7.5%, and 4% unimproved sources of water in NFHS-2, NFHS-3, NFHS-4, and NFHS-5, respectively. The percentage of unimproved type of sanitation facility was 71% in NFHS-2, which reduced to 24% in NFHS-5. Table 2 indicates that the prevalence of stunting is 44.5% for NFHS-2, 48% for NFHS-3, 38.4% for NFHS-4, and 35.5% for NFHS-5. Similarly, for wasting, the prevalence was 16%, 20%, 21%, and 19%, respectively, and for underweight, the prevalence was 47%, 43%, 36%, and 32%, respectively. The observed prevalence is adjusted by survey weights according to the DHS guideline [33], and trends are shown at the regional level in Figures 3-5. The results were consistent with the prevalence reported in the DHS reports.

**Table 1:** Sociodemographic characteristics of the samples from National Family Health Survey (NFHS)–2, NFHS-3, NFHS-4, and NFHS-5.

Sociodemographic characteristic			NFHS-2 (1998-1999), n (%)^a^		NFHS-3 (2005-2006), n (%)^a^		NFHS-4 (2015-2016), n (%)^a^		NFHS-5 (2019-2021), n (%)^a^
**Age (months)**									
	0-24	19,567 (69.9)		21,522 (41.1)		96,928 (41.1)		91,269 (41.2)	
	25-59	8402 (30.0)		30,763 (58.8)		138,783 (58.8)		129,994 (58.7)	
**Sex**									
	Male	16,996 (51.8)		29,415 (52.1)		130,572 (52.2)		119,960 (51.6)	
	Female	15,768 (48.1)		27,022 (47.8)		119,394 (47.7)		110,910 (48.0)	
**Household wealth**									
	Poor	11,619 (35.2)		27,030 (47.8)		118,332 (47.3)		117,869 (50.6)	
	Middle	10,404 (31.5)		11,180 (19.8)		49,577 (19.8)		45,083 (19.4)	
	Rich	10,990 (33.3)		18,226 (32.3)		82,056 (32.8)		69,968 (30.4)	
**Type of residence**									
	Rural	25,498 (77.8)		42,134 (74.6)		198,248 (76.3)		169,342 (73.4)	
	Urban	7266 (22.1)		14,303 (25.3)		61,379 (24.1)		61,527 (27.1)	
**Caste or tribe**									
	Scheduled caste	6556 (20.2)		11,693 (21.2)		53,851 (22.3)		53,756 (24.4)	
	scheduled tribe	3121 (9.6)		5442 (9.8)		26,350 (10.9)		23,140 (10.4)	
	Other backward classes	10,514 (32.4)		22,716 (41.2)		110,399 (45.7)		100,407 (45.5)	
	Don’t know/other	12,189 (37.6)		15,286 (27.7)		50,687 (21.0)		43,256 (20.0)	
**Mothers’ education**									
	No education	17,786 (54.3)		28,237 (50.0)		75,140 (30.1)		49,305 (21.4)	
	Primary	5159 (15.7)		7919 (14.0)		35,119 (14.1)		28,434 (12.3)	
	Secondary	7230 (22.0)		17,463 (31.0)		113,519 (45.4)		117,031 (51.1)	
	Higher	2578 (8.0)		2816 (5.0)		26,187 (10.4)		36,098 (15.2)	
**Source of drinking water**									
	Improved source	25,263 (77.1)		44,603 (86.6)		216,438 (92.4)		208,654 (90.4)	
	Unimproved source	7495 (22.8)		6884 (13.4)		17,755 (7.5)		8359 (4.0)	
**Type of sanitation facility**									
	Improved	9562 (29.2)		22,846 (40.4)		135,844 (54.34)		162,339 (70.3)	
	Unimproved (open defecation)	23,199 (70.8)		33,526 (59.4)		114,122 (45.6)		54,810 (23.7)	

^a^n (%): number of observations (proportion of observations).

**Table 2:** Prevalence of stunting, wasting, and underweight among children during different rounds of National Family and Health Survey (NFHS), India (N=577,128).

Variable		NFHS-2 (1998-1999), n (%)^a^	NFHS-3 (2005-2006), n (%)^a^	NFHS-4 (2015-2016), n (%)^a^	NFHS-5 (2019-2021), n (%)^a^
Total observations, n		33,026	51,555	259,627	232,920
Total student for height and weight		24,395 (73.8)	45,377 (88.0)	215,511 (83.0)	206,112 (88.5)
**Height for age**					
	Severely stunted	5510 (22.5)	10,758 (23.7)	34,983 (16.2)	30,439 (15.1)
	stunted	10,958 (44.9)	21,798 (48.0)	82,698 (38.4)	71,566 (35.5)
**Weight for age**					
	Severely underweight	4322 (17.7)	7185 (15.8)	24,232 (11.0)	21,885 (10.6)
	Underweight	11,391 (46.7)	19,283 (42.5)	77,005 (35.7)	66,118 (32.1)
**Weight for height**					
	Severely wasted	694 (2.8)	2916 (6.4)	16,012 (7.5)	15,144 (7.6)
	Wasted	3860 (15.7)	8991 (19.8)	45,338 (21.0)	38,024 (19.2)

^a^n (%): number of observations (proportion of observations).

### Trends of Stunting, Wasting, and Underweight

From 1998-1999 to 2005-2006, the prevalence of stunting increased in most of the regions, including Central, South, West, and Northeast, and started decreasing from 2005 onward (Figure 3). The prevalence of wasting increased from 1998 to 2015 and decreased in the subsequent 5 years (Figure 4). The prevalence of underweight decreased from NFHS-2 to NFHS-5 in all Indian regions (Figure 5).

### Spatial Autocorrelation of Stunting, Wasting, and Underweight Among Children

During the survey rounds NFHS-2 to NFHS-5, the spatial patterns of stunting, wasting, and underweight among children were found to be nonrandom. For stunting, wasting, and underweight, the value of Global Moran I varied from 0.293 to 0.419, 0.253 to 0.033, and 0.404 to 0.584, respectively, indicating significant clustering of these indicators across the country (Table 3) in NFHS-2, NFHS-4, and NFHS-5. In NFHS-4, the Moran I value was the highest, indicating considerable positive spatial autocorrelation, that is, districts with comparable prevalence are clustered together. Similarly, the spatial patterns for the source of drinking water, sanitation, wealth index, maternal education, and caste were found to be nonrandom during the survey rounds NFHS-2 to NFHS-5. The values of Global Moran I varied from 0.344 to 0.352 for the drinking water source, 0.443 to 0.459 for sanitation facility, 0.473 to 0.417 for maternal education, 0.361 to 0.472 for ethnicity, and 0.524 to 0.637 for wealth index (Table 3). The clustering trends were highly significant (>90%) during this study period. Table 4 represents the bivariate Moran I value of the outcome and explanatory variables. The highest bivariate Moran I (CGF vs explanatory variable) value was for stunting versus maternal education (−0.444), followed by stunting versus improved sanitation facility (−0.439), stunting versus wealth index (−0.426), and underweight versus improved sanitation facility (−0.487) during the NFHS-4 round.

**Table 3:** Univariate Moran I value for the outcome and explanatory variable.

Variable	Univariate Moran I (*P* value), NFHS^a^-2	Univariate Moran I (*P* value), NFHS-4	Univariate Moran I (*P* value), NFHS-5
Stunting	0.293 (<.001)	0.555 (<.001)	0.419 (<.001)
Wasting	0.253 (<.001)	0.410 (<.001)	0.033 (.10)
Underweight	0.404 (<.001)	0.659 (<.001)	0.584 (<.001)
Improved source of drinking water	0.344 (.22)	0.414 (<.001)	0.352 (<.001)
Improved sanitation facility	0.444 (.97)	0.621 (<.001)	0.459 (<.001)
Maternal education	0.459 (<.001)	0.537 (<.001)	0.417 (<.001)
Caste	0.345 (<.001)	0.438 (<.001)	0.472 (<.001)
Wealth index	0.502 (<.001)	0.640 (<.001)	0.637 (<.001)

^a^NFHS: National Family Health Survey.

**Table 4:** Bivariate Moran I values for the outcome and explanatory variable.

Variable			Bivariate Moran I (*P* value), NFHS^a^-2		Bivariate Moran I (*P* value), NFHS-4		Bivariate Moran I (*P* value), NFHS-5
**Stunting**							
	Improved source of drinking water	0.103 (.001)		0.051 (.01)		−0.016 (.01)	
	Improved sanitation facility	−0.246 (.001)		−0.439 (.01)		0.301 (.01)	
	Maternal education	0.028 (.002)		−0.444 (.01)		0.271 (.01)	
	Caste	0.000 (<.001)		−0.151 (.01)		0.125 (.01)	
	Wealth index	−0.198 (.001)		−0.426 (.01)		0.346 (.01)	
**Wasting**							
	Improved source of drinking water	−0.059 (.001)		0.055 (.001)		0.003 (.36)	
	Improved sanitation facility	−0.180 (.001)		−0.246 (.001)		0.100 (.01)	
	Maternal education	0.065 (<.001)		−0.093 (.001)		0.066 (.01)	
	Caste	−0.108 (<.001)		−0.137 (.001)		0.039 (.01)	
	Wealth index	−0.083 (<.001)		−0.098 (.001)		0.097 (.01)	
**Underweight**							
	Improved source of drinking water	0.103 (.001)		0.084 (.001)		−0.009 (.100)	
	Improved sanitation facility	−0.356 (.001)		−0.487 (.001)		0.360 (.01)	
	Maternal education	−0.259 (.001)		−0.368 (.001)		0.252 (.01)	
	Caste	−0.043 (.001)		−0.205 (.001)		0.144 (.01)	
	Wealth index	−0.169 (.001)		−0.367 (.001)		0.331(.01)	

^a^NFHS, National Family and Health Survey.

### Hotspot Detection

**Figure 6:**
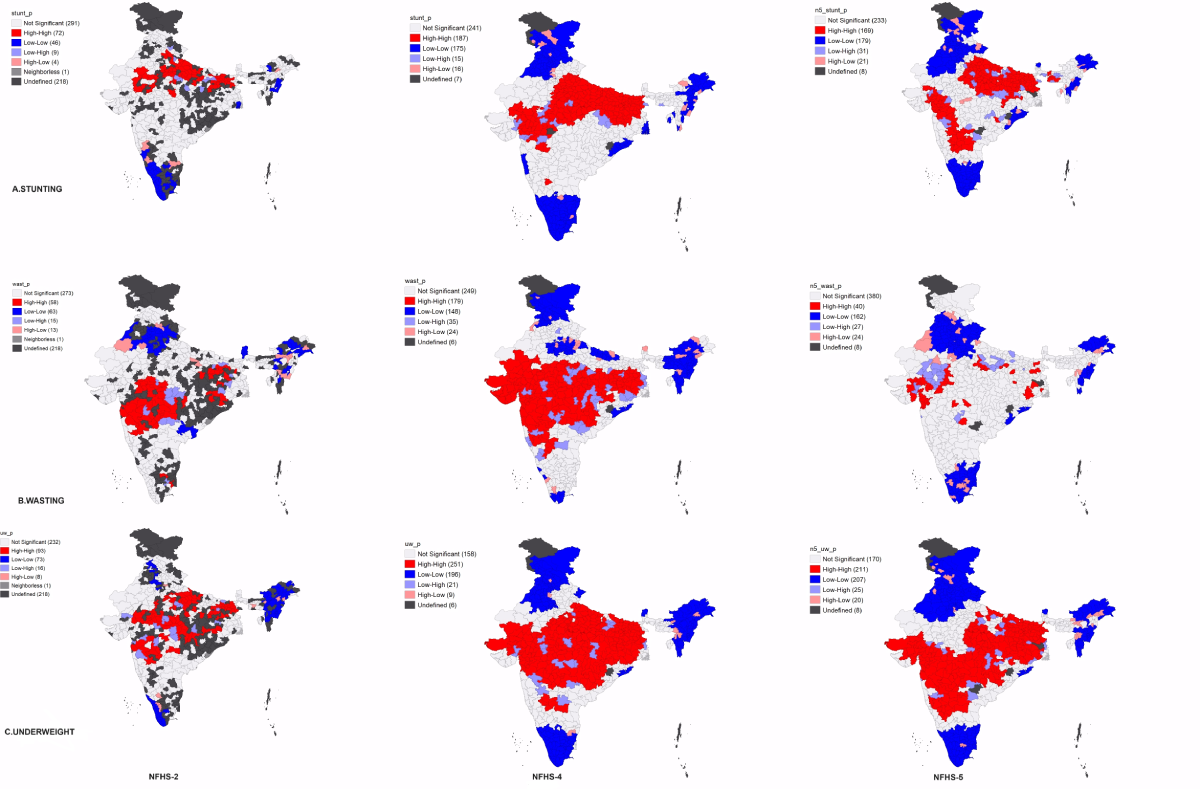
Univariate LISA (Local Indicator of Spatial Association) maps for the prevalence of (A) stunting, (B) wasting, and (C) underweight. NFHS: National Family Health Survey.

The data associated with the shapefile, along with the spatial relationship for each district-level observation, are visualized using Moron’s scatter plot. Figure 6 represents the univariate LISA maps for stunting, wasting, and underweight. The hotspot areas (enumeration areas with a high risk of stunting) were identified in districts of Rajasthan, Madhya Pradesh, Jharkhand, Bihar, Uttar Pradesh, Gujarat, West Bengal, and Chhattisgarh. Conversely, Karnataka, Arunachal Pradesh, Assam, Kerala, and Tamil Nadu were identified as the cold spots (areas with a low risk for stunting) in all the 3 surveys (NFHS-2, NFHS-4, and NFHS-5). Meanwhile, Haryana, Punjab, Himachal Pradesh, Manipur, Mizoram, Tripura, Odisha, Andhra Pradesh, Nagaland, Uttarakhand, and Maharashtra were identified as the cold spot regions in the fourth and fifth surveys. The observed blank spots on the Indian map result from a lack of data for the corresponding districts. Similarly, the hotspots for wasting were found in the districts of Maharashtra, Madhya Pradesh, Bihar, Odisha, Jharkhand, Gujarat, Andhra Pradesh, Rajasthan, Karnataka, West Bengal, and Chhattisgarh. The cold spot exists in Uttar Pradesh, Haryana, Punjab, Assam, Arunachal Pradesh, Uttarakhand, Manipur, Kerala, Nagaland, Mizoram, and Himachal Pradesh. The hotspot areas for underweight were Uttar Pradesh, Bihar, Maharashtra, Madhya Pradesh, Odisha, Jharkhand, Rajasthan, West Bengal, Andhra Pradesh, Gujarat, Karnataka, and Chhattisgarh. The cold spots were located in Kerala, Manipur, Himachal Pradesh, Punjab, Arunachal Pradesh, Tamil Nadu, Assam, Nagaland, Uttarakhand, Kerala, Mizoram, Haryana, Karnataka, and Punjab.

### LISA Modeling

**Figure 7:**
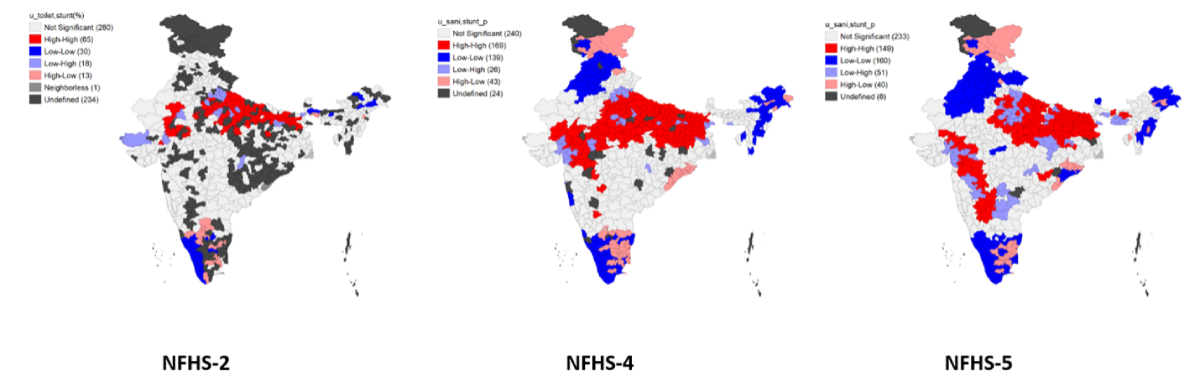
LISA (Local Indicator of Spatial Association) map showing the association between stunting and household sanitation facility between 1998 and 2021.

**Figure 8:**
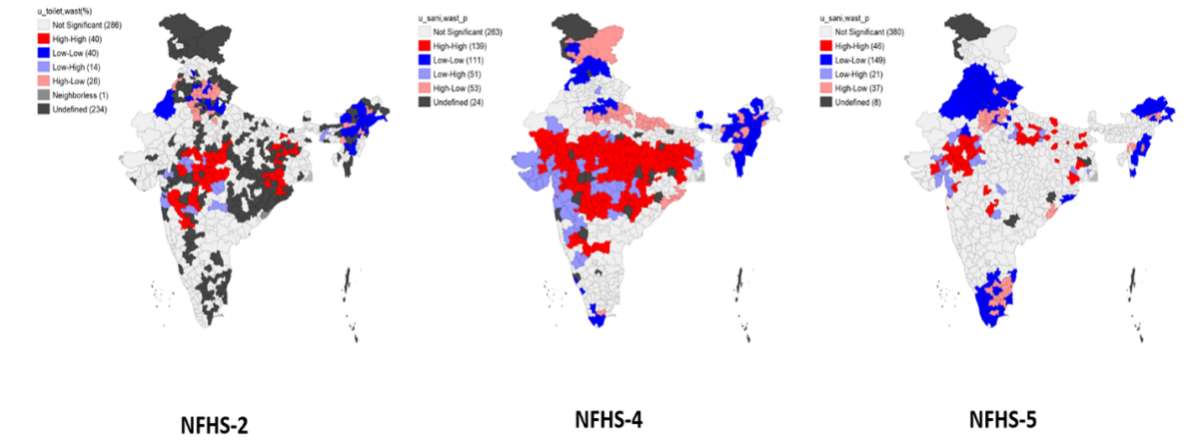
LISA (Local Indicator of Spatial Association) map showing the association between wasting and household sanitation facility between 1998 and 2021.

**Figure 9:**
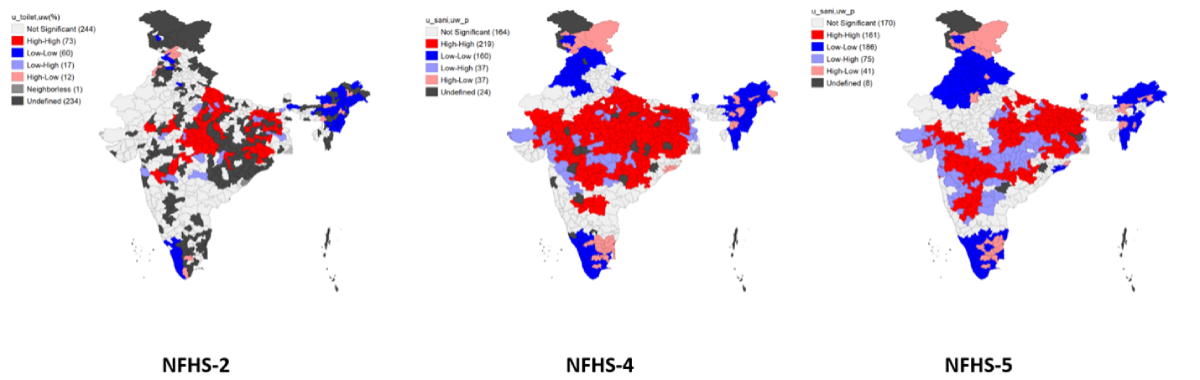
LISA (Local Indicator of Spatial Association) map showing the association between underweight and household sanitation facility between 1998 and 2021.

Figures 7-9 represent the bivariate LISA maps showing associations of stunting, wasting, and underweight with sanitation, respectively. For stunting and unimproved sanitation facilities, the hotspots are in Uttar Pradesh, Gujarat, Madhya Pradesh, Chhattisgarh, West Bengal, and Meghalaya. Regarding wasting and sanitation, Andhra Pradesh, Maharashtra, Madhya Pradesh, Gujarat, Rajasthan, West Bengal, and Chhattisgarh are the hotspot areas. Similarly, for underweight, Maharashtra, West Bengal, Jharkhand, Uttar Pradesh, Madhya Pradesh, Andhra Pradesh, Gujarat, Chhattisgarh, and Rajasthan are high-risk areas.

Hotspots for stunting and unimproved drinking water sources are in the districts of Rajasthan, Gujarat, Madhya Pradesh, Bihar, West Bengal, Uttar Pradesh, and Maharashtra. For wasting, hotspots are in Gujarat, Maharashtra, Rajasthan, Andhra Pradesh, Karnataka, Madhya Pradesh, Uttar Pradesh, West Bengal, Chhattisgarh, Bihar, and Odisha. The common hotspots for underweight and unimproved drinking water sources are the districts of Uttar Pradesh, Bihar, Chhattisgarh, Andhra Pradesh, Jharkhand, Maharashtra, Gujarat, Karnataka, Madhya Pradesh, Odisha, West Bengal, and Rajasthan. The hotspots for maternal education and CGF indicators are in Jharkhand, Gujarat, Uttar Pradesh, Chhattisgarh, Maharashtra, Madhya Pradesh, West Bengal, and Rajasthan for stunting. For wasting, these areas include Maharashtra, West Bengal, Karnataka, Chhattisgarh, Gujarat, Jharkhand, Odisha, Madhya Pradesh, and Rajasthan. The hotspots for underweight are in Gujarat, Maharashtra, Uttar Pradesh, Odisha, West Bengal, Chhattisgarh, Madhya Pradesh, Rajasthan, and Jharkhand. The hotspots for poor wealth index and CGF indicators are in Bihar, Uttar Pradesh, Gujarat, West Bengal, Madhya Pradesh, and Maharashtra for stunting. The hotspots for wasting are in Andhra Pradesh, Maharashtra, Madhya Pradesh, Rajasthan, Gujarat, and Karnataka. For underweight, Maharashtra, Rajasthan, Madhya Pradesh, and Chhattisgarh are consistently high-risk areas from 1998 to 2006. Uttar Pradesh and Gujarat are the hotspots added during the fourth and fifth rounds only. The hotspot areas for stunting and ethnic groups are Madhya Pradesh, Rajasthan, Jharkhand, West Bengal, Gujarat, Uttar Pradesh, Bihar, and Maharashtra. Similarly, for wasting, the hotspot areas are Uttar Pradesh, Gujarat, Mizoram, Rajasthan, Maharashtra, Kerala, Uttarakhand, Punjab, Andhra Pradesh, Jammu & Kashmir, Odisha, Assam, Tamil Nadu, Manipur, Meghalaya, Karnataka, Bihar, and West Bengal. The underweight hotspots are Uttar Pradesh, Rajasthan, Bihar, Jharkhand, Gujarat, West Bengal, Andhra Pradesh, Karnataka, Odisha, Maharashtra, and Madhya Pradesh.

The map’s red areas (hotspots) are concerning areas for stunting, wasting, and underweight. Uttar Pradesh, Odisha, Bihar, Rajasthan, Chhattisgarh, Madhya Pradesh, West Bengal, Gujarat, and Jharkhand are some of the hotspot areas for wasting, stunting, and underweight and their background characteristics. Tamil Nadu, Assam, Arunachal Pradesh, Manipur, Meghalaya, and Nagaland are the cold spots for wasting, stunting, and underweight and background characteristics throughout all the surveys. Haryana, Punjab, Himachal Pradesh, Mizoram, Karnataka, Uttarakhand, and Tripura are the states whose indicators for both the dependent and independent variables have seen to improve in the NFHS-4 and NFHS-5 rounds.

### Spatial Regression

The test for multicollinearity suggests that there are unnecessary dependences between the explanatory variables in the data from NFHS-2 and NFHS-4. The Breusch-Pagan test indicates the presence of heteroskedasticity (*P*<.001), and the Jarque-Bera test reveals a violation of the assumption of normal distribution (*P*<.001). The rejection of these assumptions suggests that the data are spatially dependent, which necessitates the use of spatial regression. Compared with the lag model, the spatial error model demonstrated a lower Akaike information criterion score for NFHS-2, NFHS-4, and NFHS-5, indicating a better fit for the data from these surveys.

### Stunting

Table 5 presents the regression coefficients for the association between stunting and sanitation facility, obtained from both ordinary least-squares (OLS) and the spatial error model. According to the models for NFHS-2, NFHS-4, and NFHS-5, with each unit increment in sanitation facility (%), wealth quintile (%), and mothers’ education (%), the prevalence of stunting is expected to decrease by 0.109-0.145, 0.049-0.111, and 0.058-0.274 units, respectively.

**Table 5:** Regression coefficients for stunting obtained from the ordinary least-squares (OLS) and the spatial error model (SEM)^a^.

	Year/Variable	OLS model				SEM		
		Coefficient	SE	*P* value	Coefficient		SE	*P* value
**1998-1999**								
	Constant	65.778	2.447	.001	66.425		2.665	.001
	Water facility	0.002	0.025	.10	−0.002		0.026	.92
	Sanitation	−0.117	0.026	.001	−0.109		0.029	.001^b^
	Wealth	−0.069	0.026	.01	−0.069		0.029	.02^b^
	Education	−0.271	0.033	.001	−0.274		0.036	.001^b^
	Caste	0.006	0.024	.10	−0.009		0.026	.72
	Lambda	—^c^	—	—	0.353		0.056	.001^b^
**2015-2016**								
	Constant	51.114	2.378	.001	47.165		2.538	.001
	Water facility	0.101	0.022	.001	0.087		0.024	<.001^b^
	Sanitation	−0.110	0.017	.001	−0.145		0.021	.001^b^
	Wealth	−0.071	0.018	.001	−0.049		0.022	.03^b^
	Education	−0.178	0.020	.001	−0.101		0.023	.001^b^
	Caste	−0.040	0.013	.01	−0.035		0.015	.02^b^
	Lambda	—	—	—	0.643		0.037	.001
**2019-2021**								
	Constant	45.742	3.629	.001	45.348		3.707	.001
	Water facility	0.132	0.033	.001	0.086		0.035	.02^b^
	Sanitation	−0.138	0.023	.001	−0.117		0.025	.001^b^
	Wealth	−0.103	0.014	.001	−0.111		0.017	.001^b^
	Education	−0.099	0.020	.001	−0.058		0.021	.007^b^
	Caste	−0.034	0.014	.02	−0.030		0.017	.09
	Lambda	—	—	—	0.484		0.046	.001

^a^Akaike information criterion of spatial lag model (SLM)>SEM: National Family and Health Survey (NFHS)-2: 3160.3 (SLM), 3145.7 (SEM); NFHS-4: 3986.2 (SLM), 3950.1 (SEM); and NFHS-5: 4207.1 (SLM), 4207.1 (SEM).

^b^*P*<.05 denotes a significant impact on stunting prevalence.

^c^Not available.

### Wasting

Table 6 represents the regression coefficients for the association between wasting and sanitation facility, as obtained from OLS and the spatial error model. In all 3 models, a unit increase in sanitation facility (%) results in an expected decrease in the prevalence of wasting by 0.073 to 0.266 units.

**Table 6:** Regression coefficients for wasting obtained from the ordinary least-squares (OLS) and the spatial error model (SEM)^a^.

	Year/Variable	OLS model				SEM		
		Coefficient	SE	*P* value	Coefficient		SE	*P* value
**1998-1999**								
	Constant	65.778	2.447	.001	66.425		2.151	<.001
	Water facility	0.002	0.025	.100	−0.002		0.021	.95
	Sanitation	−0.117	0.026	.001	−0.109		0.023	<.001^b^
	Wealth	−0.069	0.026	.01	−0.069		0.024	.81
	Education	−0.271	0.033	.001	−0.274		0.029	.81
	Caste	0.006	0.024	.10	−0.009		0.021	.001^b^
	Lambda	—^c^	—	—	0.353		0.058	<.001
**2015-2016**								
	Water facility	−0.020	0.024	.10	−0.052		0.026	.04^b^
	Sanitation	−0.138	0.018	.001	−0.073		0.022	.001^b^
	Wealth	0.050	0.019	.001	0.014		0.023	.54
	Education	−0.002	0.022	.10	−0.034		0.024	.16
	Caste	−0.026	0.014	.05	−0.009		0.016	.57
	Lambda	—	—	—	0.633		0.038	<.001
**2019-2021**								
	Constant	25.631	11.391	.02^b^	25.698		11.297	.02
	Water facility	0.079	0.105	.45	0.079		0.104	.45
	Sanitation	−0.266	0.074	<.001^b^	−0.266		0.073	<.001^b^
	Wealth	−0.051	0.045	.26	−0.051		0.044	.25
	Education	0.099	0.065	.13	0.098		0.064	.13
	Caste	0.019	0.046	.67	0.019		0.046	.68
	Lambda	—	—	—	−0.011		0.063	.86

^a^Akaike information criterion of spatial lag model (SLM)>SEM: National Family and Health Survey (NFHS)-2: 2979 (SLM), 2978 (SEM); NFHS-4: 4029 (SLM), 4021 (SEM); and NFHS-5: 5661.7 (SLM), 5661.6 (SEM).

^b^*P*<.05 denotes a significant impact on stunting prevalence.

^c^Not available.

### Underweight

Table 7 represents the regression coefficients for the association between underweight and sanitation facility, as obtained from both OLS and the spatial error model. For each unit increase in sanitation facility (%), the decrease of underweight is expected to decrease by 0.124 to 0.157 units.

**Table 7:** Regression coefficients for underweight obtained from the ordinary least-squares (OLS) and the spatial error model (SEM)^a^.

	Year/Variable	OLS model				SEM		
		Coefficient	SE	*P* value	Coefficient		SE	*P* value
**1998-1999**								
	Constant	63.229	2.502	<.001	64.817		2.734	<.001
	Water facility	0.006	0.026	.10	0.003		0.026	.88
	Sanitation	−0.209	0.027	<.001	−0.124		0.030	.001^b^
	Wealth	−0.058	0.026	.05	−0.061		0.031	.05^b^
	Education	−0.160	0.034	<.001	−0.223		0.036	.001^b^
	Caste	−0.022	0.024	.10	−0.053		0.026	.04^b^
	Lambda	—^c^	—	—	0.462		0.042	<.001
**2015-2016**								
	Constant	27.756	2.514	<.001	48.083		2.728	<.001
	Water facility	−0.020	0.024	.10	0.011		0.025	.65
	Sanitation	−0.138	0.018	.001	−0.157		0.023	<.001^b^
	Wealth	0.050	0.019	.001	−0.025		0.023	.28
	Education	−0.002	0.022	.10	−0.095		0.024	<.001^b^
	Caste	−0.026	0.014	.05	0.000		0.016	.96
	Lambda	—	—	—	0.788		0.027	<.001
**2019-2021**								
	Constant	48.481	4.237	<.001	43.869		3.551	<.001
	Water facility	0.103	0.039	.001	0.006		0.033	.85
	Sanitation	−0.273	0.027	.001	−0.137		0.025	.001^b^
	Wealth	−0.081	0.016	.001	−0.104		0.018	.001^b^
	Education	−0.044	0.024	.10	0.010		0.020	.61
	Caste	−0.040	0.017	.01	−0.011		0.018	.56
	Lambda	—	—	—	0.759		0.029	<.001

^a^Akaike information criterion of spatial lag model (SLM)>SEM: National Family and Health Survey (NFHS)-2: 3153.1 (SLM), 3127.6 (SEM); NFHS-4: 4027.9 (SLM), 3988.9 (SEM); and NFHS-5: 4093 (SLM), 4052.8 (SEM).

^b^*P*<.05 denotes a significant impact on stunting prevalence.

^c^Not available.

## Discussion

### Principal Findings

This study used LISA modeling to establish a significant association between improved sanitation facilities and the reduction of growth failure health outcomes in children younger than 5 years in India. The relative risk maps identified the hotspots of CGF health cases, which could be targeted for future interventions. Nutritional status is critical for monitoring a country’s development. Moreover, undernutrition is a global concern that poses numerous health, welfare, and well-being challenges [5]. Consequently, it becomes a pressing matter in the context of Sustainable Development Goals. Although global efforts have intensified over the past decade, governments have also strategized to address undernutrition. In addition, research has sought to unravel potential impediments that could lead to health and economic problems in developing countries. Although these issues were primarily associated with inadequate feeding patterns [12], this remains insufficient to address the problem, especially in India where undernutrition affects both the well-nourished and malnourished [13]. Researchers have striven to examine various factors that retard children’s growth, but clear evidence linking it to sanitation, particularly unimproved drinking water sources facilities (open defecation), is yet to emerge.

According to this study’s findings, more than a third of children younger than 5 years are malnourished. A study comparing the nutritional health of Indian children using WHO charts and an Indian synthetic chart also discovered a higher prevalence of undernutrition among this age group [34]. Neighboring countries such as Nepal (36.0%) [35] and Pakistan (37.6%) [36] have nearly identical stunting rates, whereas Bangladesh (18.2%) [37] has comparable wasting and underweight rates. Our study shows a decreasing trend in stunting (44.9% to 38.4%) and underweight (46.7% to 35.7%) but an increasing prevalence of wasting (15.7% to 21.0%) over 15 years. Since 1990, despite a decrease in the prevalence of undernutrition in India, it has been a significant concern. The Global Burden of Disease Study (1990-2017) stated that malnutrition remained the leading risk factor for disease burden in India and that much faster progress is needed to meet India’s 2022 and global 2030 targets [14].

The spatial findings show that the CGF and water, sanitation, and hygiene (WaSH) indices are nonrandom at the national and district levels. Significant clusters for these indicators were found in several states. However, during the NFHS-4 survey, indicators improved for both factors in states such as Haryana, Punjab, Himachal Pradesh, Manipur, Mizoram, Karnataka, Uttarakhand, and Tripura. Many factors, ranging from demographic to socioeconomic, contribute to the increasing prevalence of undernutrition, which can be attributed to regional preferences in food consumption and disparities in the availability of health care facilities [38]. Other factors could be inadequate sanitation and lack of clean water, which elevate the prevalence of soil-transmitted diseases, such as diarrhea, contributing to undernutrition. This finding aligns with a study that found significant geographical variations across in child malnutrition across Indian states, with particularly high rates in the central region [39].

The measure of spatial dependence was suggested by Moran I statistics. Stunting, underweight, and toilet facility have higher statistical values, confirming the regional gradient of malnutrition and sanitation in India. The results of the spatial error model show that increased sanitation and the level of mothers’ education are strongly and significantly associated with child malnutrition. As previously noted, poor sanitation can lead to diarrhea and other intestinal infections in children. Furthermore, the positive correlation between child nutrition and a mother’s education status aligns a previous study [40] that found a connection between maternal education and improved nutrition [41]. The findings of this study concerning the spatial relationship and correlation with WaSH factors are comparable to those of an Indian study using NFHS-4 data. Similar to the findings of this study, that study found a clear spatial dependence of the CGF indicator and increased clustering in the districts of Madhya Pradesh, Rajasthan, Bihar, Jharkhand, and Uttar Pradesh. There was also a statistically significant link between malnutrition and maternal education, improved sanitation, and wealth [42]. However, our findings reveal a strong link to WaSH variables, which, according to UNICEF, are important contributors to malnutrition [43]. Several studies have found that a lack of sanitary facilities increases the risk of nutritional deficiencies due to anorexia, fluids loss, and other complications that can lead to undernutrition [44]. Similarly, a study conducted in Ethiopia found that children living in households where open pit or unsanitary pit latrines were used were more likely to have malnutrition [45].

In 2014, the Indian government launched a cleanliness initiative called “Swachh Bharat Abhiyan” to enhance waste management and sanitation quality across the country, aligning with the sanitation aspect of child nutrition [46]. The multifaceted causes of undernutrition necessitate coordinated and consistent interventions that go beyond the norm. Exposure to fecal pathogens poses a significant threat to children’s growth, making improved WaSH conditions essential in all settings [47]. This could be achieved by embracing various aspects of WaSH practices, particularly incorporating sanitation into the nutrition program, strengthening the collaboration between WaSH and nutrition factors, and making WaSH initiatives more nutrition sensitive.

### Strength and Limitations

Given the widespread socioeconomic and regional disparities among people in India, this study provides estimates of the prevalence of stunting, wasting, and underweight at the district level. The study’s regional spatial approach will assist policy makers in developing policies that ensure district-specific interventions for improving malnutrition. Moreover, the study’s results can be well generalized by using a nationally representative sample from a well-known large-scale survey in India. However, the study also has some limitations. First, because of the cross-sectional nature of the data, we could not establish a causal relationship among the variables. Second, because of the unavailability of NFHS-3 district-level data, a spatial analysis for NFHS-3 could not be conducted. Third, as the study relied on secondary data, we were unable to investigate community-level factors such as the availability of improved drinking water sources and sanitation facilities that might influence sanitation practice behavior among women. These factors could be investigated more deeply in future qualitative studies.

### Conclusions

This study underscores that the malnutrition and undernutrition remain concerns in India. The prevalence of stunting does not seem to be decreasing or increasing consistently. However, wasting and underweight show increasing and decreasing trends, respectively. This study demonstrates that the presence of improved sanitation facilities and the educational status of mothers have a substantial positive correlation with the risk of CGF indicators. Policy makers can incorporate these factors when devising specific interventions to improve malnutrition indicators. The implementation of WaSH interventions should aim to save women’s time; if the time spent fetching water can be reduced, women could devote more time to childcare. Moreover, lack of access to WaSH facilities impacts the academic success of school-aged children, resulting in a decreased likelihood of securing employment, household food insecurity, and perpetuated poverty—all primary factors of child malnutrition. On the other hand, improved sanitation facilities have positive socioeconomic outcomes, such as supporting women’s dignity and safety, increasing girls’ school attendance, and facilitating nutrient recovery.

## Abbreviations

CGF: child growth failure
DHS: Demographic and Health Survey
LISA: Local Indicator of Spatial Association
NFHS: National Family Health Survey
OLS: ordinary least squares
WaSH: water, sanitation, and hygiene
WHO: World Health Organization
